# Development and validation of a nomogram to assess postoperative venous thromboembolism risk in patients with stage IA non‐small cell lung cancer

**DOI:** 10.1002/cam4.4982

**Published:** 2022-06-27

**Authors:** Yongsheng Cai, Honghong Dong, Xinyang Li, Yi Liu, Bin Hu, Hui Li, Jinbai Miao, Qirui Chen

**Affiliations:** ^1^ Department of Thoracic Surgery Beijing Institute of Respiratory Medicine and Beijing Chao‐Yang Hospital, Capital Medical University Beijing China

**Keywords:** non‐small cell lung cancer, stage IA, venous thromboembolism, nomogram, caprini risk assessment model

## Abstract

**Background:**

Venous thromboembolism (VTE) is a common postoperative complication in patients with lung cancer that seriously affects prognosis and quality of life. At present, the detection rate of patients with early‐stage lung cancer is increasing, but there are few studies on the risk factors for postoperative venous thromboembolism (VTE) in patients with stage IA non‐small cell lung cancer (NSCLC). This study aimed to establish a nomogram for predicting the probability of postoperative VTE risk in patients with stage IA NSCLC.

**Methods:**

The clinical data of 452 patients with stage IA NSCLC from January 2017 to January 2022 in our center were retrospectively analyzed and randomly divided into a training set and a validation set at a ratio of 7:3. Independent risk factors were identified by univariate and multivariate logistic regression analyses, and a nomogram was established based on the results and internally validated. The predictive power of the nomogram was evaluated by receiver operating characteristic curve (ROC), calibration curve, and decision curve analysis (DCA).

**Results:**

The nomogram prediction model included three risk factors: age, preoperative D‐dimer, and intermuscular vein dilatation. The areas under the ROC curve of this predictive model were 0.832 (*95% CI*: 0.732–0.924) and 0.791 (*95% CI*: 0.668–0.930) in the training and validation sets, respectively, showing good discriminative power. In addition, the probability of postoperative VTE occurrence predicted by the nomogram was consistent with the actual occurrence probability. In the decision curve, the nomogram model had a better net clinical benefit at a threshold probability of 5%–90%.

**Conclusion:**

This study is the first to develop a nomogram for predicting the risk of postoperative VTE in patients with stage IA NSCLC; this nomogram can accurately and intuitively evaluate the probability of VTE in these patients and help clinicians make decisions on prevention and treatment.

## Graphical abstract

In order to identify patients with high risk of venous thromboembolism after surgery for stage IA non‐small cell lung cancer earlier, we development and validation of a nomogram. Our study confirmed that the model has good discriminative ability.

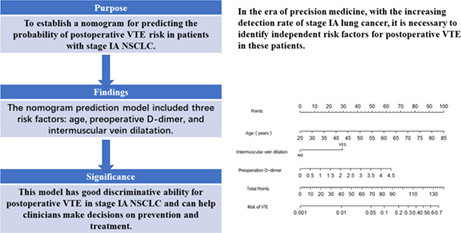

## 1 INTRODUCTION

Venous thromboembolism (VTE), which mainly includes deep venous thrombosis (DVT) and pulmonary thromboembolism (PE), is one of the most common complications of malignant tumors. Compared with the general population, cancer patients have a significantly increased risk of VTE,^1^, ^2^especially lung cancer, which currently has the highest incidence of VTE.^3^, ^4^In addition, surgical resection undoubtedly increases the probability of VTE in patients during lung cancer treatment. The occurrence of postoperative VTE events affects the treatment of the primary disease and significantly increases the risk of death of patients.^5^, ^6^

According to previous studies, the incidence of VTE after lung cancer surgery is as high as 8.1%–23.1%.^7^, ^8^, ^9^ Therefore, we need to pay attention to the early prevention and diagnosis of thrombosis in patients after surgery. At present, to identify the high‐risk groups of VTE as early as possible, a variety of VTE risk assessment models (RAM) have been proposed, including Caprini RAM,^10^ Padua RAM,^11^ Khorana RAM,^12^ Rogers RAM,^13^ etc. Different risk assessment models are used for different populations. Among them, the modified Caprini RAM^10^ is widely used in thoracic surgery and has a good stratification effect. However, with the widespread application of low‐dose, high‐resolution spiral CT in the screening of lung cancer and novel coronavirus pneumonia in recent years,^14^ the detection rate of early‐stage lung cancer has increased significantly, resulting in a great change in the characteristics of the lung cancer population in current thoracic surgery. Whether the modified Caprini risk assessment model is equally applicable to patients with early‐stage lung cancer is unclear. In our center, we previously conducted a preliminary study on VTE incidence after surgery for stage IA NSCLC and reported that the number of lymph nodes removed was an independent risk factor.^15^ However, in the era of precision medicine, it is essential to identify additional high‐risk factors for VTE in early‐stage lung cancer patients, especially in patients with stage IA lung cancer, and it is urgent to establish an accurate postoperative VTE prediction model.

A nomogram is an intuitive and visual prediction tool that can accurately predict each patient's risk probability of clinical events.^16^, ^17^, ^18^ A nomogram can make the prediction model more targeted by adding useful potential biomarkers, thereby achieving precise and personalized treatment. Nomograms have been applied to studies of various malignancies and have proven to be a reliable tool for predicting cancer prognosis.^19^, ^20^ In addition, its accuracy has been confirmed in predicting postoperative VTE of gynecological tumors, spinal metastatic tumors, and breast cancer.^17^, ^21^, ^22^However, there is currently a lack of accurate predictive models for postoperative VTE in stage IA NSCLC. Therefore, this study attempts to establish a nomogram graph model to predict the risk probability of postoperative VTE in patients with stage IA NSCLC. Through this model, clinicians can accurately identify patients with a high risk of postoperative VTE and can vividly show the probability of postoperative VTE to patients.

## 2 MATERIALS AND METHODS

### 2.1 Patient enrollment

We retrospectively analyzed 452 patients with stage pIA lung cancer who underwent lung resection at our center from January 2017 to January 2022. Inclusion criteria: (1) Primary NSCLC with pathological stage IA; (2) no VTE before surgery; and (3) complete clinical data. Exclusion criteria: (1) Patient refused surgical treatment; (2) benign lesions or nonstage IA NSCLC; (3) VTE before surgery; (4) perioperative use of anticoagulant drugs; (5) presence of hematological diseases; and (6) no lower extremity vascular ultrasound examination was performed after operation, or other data were missing. All patients were pathologically staged according to the eighth edition of lung cancer staging published by the American Joint Committee on Cancer/International Union Against Cancer.^23^ The follow‐up period ended when the patient was discharged.

### 2.2 Outcome and variables

The outcome variable in this study was the occurrence of VTE events before discharge in patients with stage IA NSCLC after surgery. All patients in our study underwent lower extremity vascular ultrasound examinations by two professional sonographers before and after the operation to determine whether there was DVT. Computed tomography pulmonary angiography (CTPA) should be performed to determine whether PE exists if the patient has typical symptoms of PE (chest pain, hemoptysis, dyspnea, or persistent hypoxemia), a high Caprini score (≥9), or a newly diagnosed postoperative DVT.

The following data were recorded through the electronic medical record system: basic information included age, sex, length of hospital stay, body mass index (BMI); surgical information included surgical approach, the extent of resection, operation time, bleeding, and the number of lymph nodes removed; coagulation function indicators included platelet (PLT), activated partial thromboplastin time (APTT), prothrombin time (PT), and D‐dimer levels preoperatively and postoperatively; preoperative pulmonary function indicators included forced exhalation in the first, second volume (FEV1), forced vital capacity (FVC), and maximum ventilation volume (MVV); imaging information included nodule morphology, nodule location, and intermuscular vein dilation (IVD); and pathological information included pathological diameter, pathological type, and T stage.

### 2.3 Nomogram construction and validation

All enrolled patients were randomly divided into a training set (*n* = 318) and a validation set (*n* = 134) at a ratio of 7:3 by computer. This ratio can ensure the maximum utilization of samples and make the validation set have a sufficient sample size. Previous studies have confirmed this ratio.^21^, ^24^, ^25^

Univariate and multivariate logistic regression analyses were used in the training set to determine the independent risk factors for postoperative VTE, and finally, a nomogram was constructed based on the factors screened out by the multivariate logistic regression results. The area under the curve (AUC) and C‐index were used to discriminate the accuracy of the model for distinguishing between VTE and non‐VTE patients. The calibration curve was applied to describe the consistency between the predicted probability and the actual probability. The closer the two lines are, the closer the predicted incidence rate is to the actual incidence rate, indicating that the nomogram prediction model has a better consistency. Finally, decision curve analysis (DCA) was used to evaluate the net benefit of patients and clinical utility by quantifying the net benefit at different threshold probabilities in the validation set.

### 2.4 Statistical analyses

All continuous variables were compared between groups using *t*‐test and are described as the mean ± standard deviation. All categorical variables were compared between groups using the chi‐square test and are described as proportions. Receiver operating characteristic (ROC) curves were plotted by the “pROC” R package. Nomograms were constructed using the “rms” R package, and calibration plots were drawn using the “rms” R package using 1000 bootstrap resampling and obtained C‐index. Clinical decision curves were drawn using the “rmda” package. Statistical analysis was performed using IBM SPSS Statistics 26.0 and R 4.0.3 (version 4.0.3; http://www.Rproject.org), and a two‐sided *p* value <0.05 was considered significant.

## 3 RESUITS

### 3.1 Patient characteristics

**FIGURE 1:**
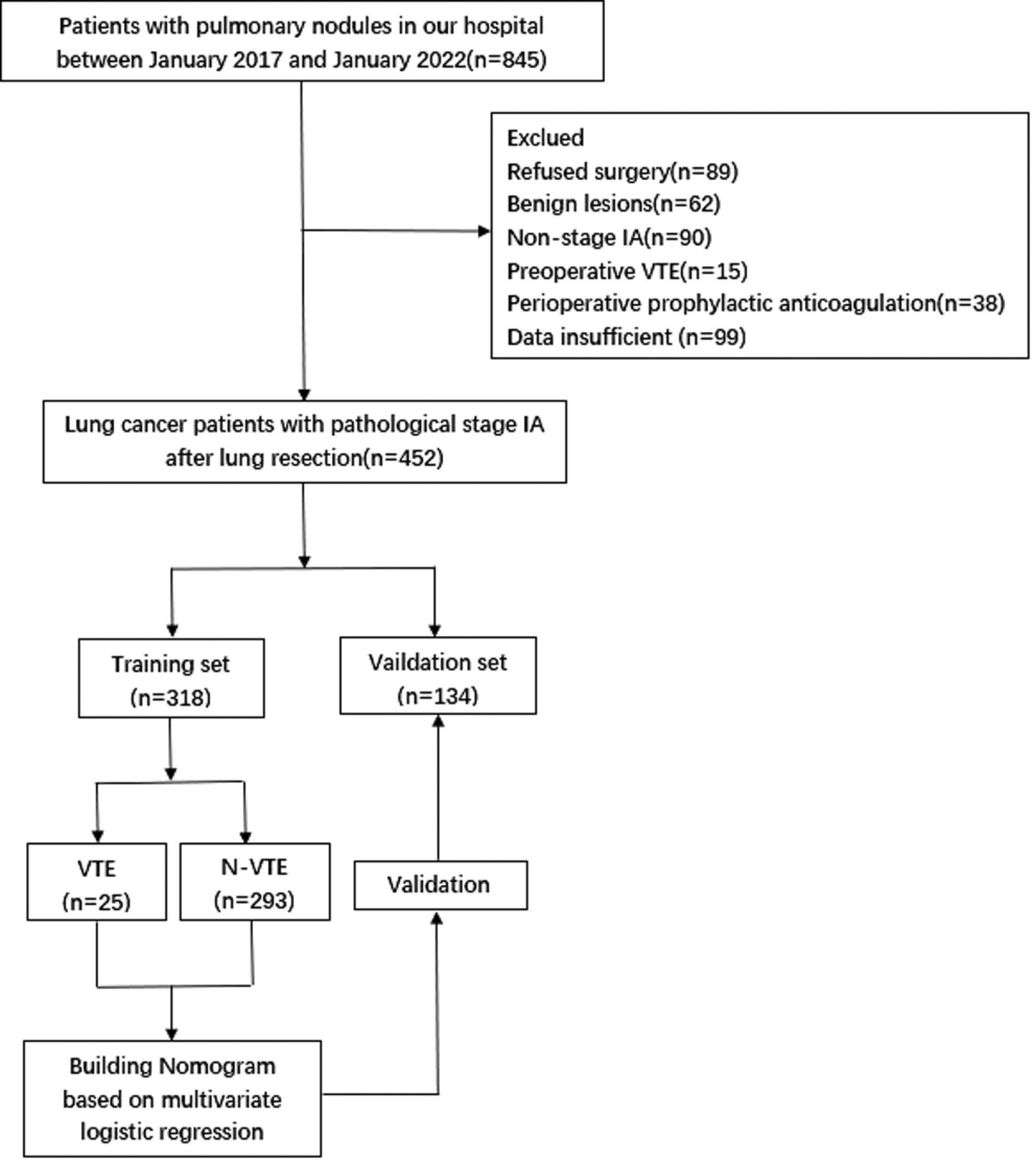
Flow chart of patient selection.VTE, venous thromboembolism

A total of 452 patients were finally included, including 179 males and 273 females, with an average age of 57.86 ± 10.15 years. Forty (8.9%) patients had postoperative VTE events, all of which were DVTs. Patients were randomly divided into 318 patients (70%) in the training set and 134 patients (30%) in the validation set at a ratio of 7:3. The screening process is shown in the flowchart (Figure 1). Except for the extent of resection (*p* = 0.027), pathological diameter (*p* = 0.015), and pathological type (*p* = 0.033), the other characteristics of the two groups were comparable, and there was no significant difference in distribution. The demographic and clinicopathological characteristics of the two groups are shown in Table 1.

**TABLE 1:** Demographics and clinicopathologic characteristics of the training and validation set

		Total	Training set	Validation set	*P*‐value
Variable		(*N* = 452)	(*N* = 318)	(*N* = 134)	
VTE		40	25 (62.5%)	15 (37.5%)	0.278
Age		57.86 ± 10.15	57.77 ± 10.15	58.05 ± 10.17	0.790
Sex					0.822
Male		179	127 (70.9%)	52 (29.1%)	
Female		273	191 (70%)	82 (30%)	
Hospital length of stay (d)		9.56 ± 4.54	9.59 ± 4.75	9.48 ± 4.01	0.813
BMI		24.41 ± 3.33	24.50 ± 3.26	24.20 ± 3.50	0.386
Surgical approach					1.000
VATS		446	314 (70.4%)	132 (29.6%)	
Open		6	4 (66.7%)	2 (33.3%)	
Extent of resection					0.027
Wedge		76	62 (81.6%)	14 (18.4%)	
Segment		67	50 (74.6%)	17 (25.4%)	
Lobe		309	206 (66.7%)	103 (33.3%)	
Operation time		129.16 ± 53.61	128.41 ± 55.91	130.95 ± 47.87	0.646
Bleeding		88.57 ± 169.86	90.14 ± 166.38	84.80 ± 178.50	0.761
Pre‐PLT		226.62 ± 59.56	227.72 ± 60.42	224.00 ± 57.59	0.545
Pre‐APTT		26.07 ± 5.44	26.28 ± 6.23	25.57 ± 2.69	0.208
Pre‐PT		11.68 ± 0.64	11.70 ± 0.63	11.64 ± 0.67	0.369
Pre‐D‐Dimer		0.35 ± 0.49	0.35 ± 0.46	0.36 ± 0.55	0.835
Post‐PLT		213.07 ± 52.70	212.81 ± 53.54	213.69 ± 50.84	0.873
Post‐APTT		25.48 ± 3.02	25.61 ± 3.00	25.15 ± 3.05	0.138
Post‐PT		12.10 ± 0.69	12.09 ± 0.70	12.10 ± 0.70	0.915
Post‐D‐Dimer		1.45 ± 1.99	1.44 ± 2.13	1.47 ± 1.59	0.912
FEV1		2.61 ± 0.65	2.59 ± 0.65	2.65 ± 0.64	0.435
FVC		3.43 ± 0.79	3.42 ± 0.80	3.45 ± 0.77	0.701
MVV		106.97 ± 29.18	106.23 ± 28.47	108.81 ± 30.93	0.407
IVD		69	50 (72.5%)	19 (27.5%)	0.667
Module morphology					0.377
GGO		158	117 (74.1%)	41 (25.9%)	
Subsolidity		158	110 (69.6%)	48 (30.4%)	
Solidity		135	90 (66.7%)	45 (33.3%)	
Location					0.561
LU		111	85 (76.6%)	26 (23.4%)	
LL		63	42 (66.7%)	21 (33.3%)	
RU		165	113 (68.5%)	52 (31.5%)	
RM		35	25 (71.4%)	10 (28.6%)	
RL		78	53 (67.9%)	25 (32.1%)	
Pathological diameter		1.37 ± 0.62	1.32 ± 0.59	1.48 ± 0.66	0.015
T stage					0.158
T1a		188	140 (75.2%)	48 (25.5%)	
T1b		212	146 (68.9%)	66 (31.1%)	
T1c		52	32 (61.5%)	20 (38.5%)	
Pathological type					0.033
AD		432	308 (71.3%)	124 (28.7%)	
SCC		18	8 (44.4%)	10 (55.6%)	
Others		2	2 (100%)	0 (0%)	
LNR		11.13 ± 8.15	10.69 ± 8.00	12.19 ± 8.45	0.082

Abbreviations: AD, adenocarcinoma; APTT, activated partial thromboplastin time; BMI, body mass index; FEV1, forced expiratory volume in one second; FVC, forced vital capacity; GGO, ground‐glass opacity; LL, left lower lobe; LNR, lymph node removal; LU, left upper lobe; MVV, maximal voluntary ventilation; PLT, platelet; PT, Prothrombin time; RL, right lower lobe; RM, right middle lobe; RU, right upper lobe; SCC, squamous cell carcinoma; VATS, video‐assisted thoracoscopic surgery; VTE, venous thromboembolism.

### 3.2 Independent risk factors for VTE in the training set

The univariate logistic analysis results shown in Table 2 showed that age (OR = 1.120, 95% CI: 1.060–1.191, *p* < 0.001), thoracotomy (OR = 12.652, 95% CI: 1.703–93.995, *p* = 0.013), operation time (OR = 1.010, 95% CI: 1.003–1.017, *p* = 0.003), blood loss (OR = 1.002, 95% CI: 1.000–1.003, *p* = 0.023), number of lymph nodes removed (OR = 1.061, 95% CI: 1.013–1.112, *p* = 0.012), preoperative D‐dimer (OR = 2.676, 95% CI: 1.456–4.919, *p* = 0.002), postoperative D‐dimer (OR = 1.154, 95% CI: 1.022–1.304, *p* = 0.021), arteriovenous vein dilatation (OR = 7.466, 95% CI: 3.169–17.592, *p* < 0.001), and T stage (OR = 3.519, 95% CI: 1.039–11.917, *p* = 0.043) were significantly positively correlated with postoperative VTE in patients with stage IA NSCLC. However, forced expiratory volume in 1 s (FEV1) (OR = 0.478, 95% CI: 0.239–0.957, *p* = 0.037), forced vital capacity (FVC) (OR = 0.554, 95% CI: 0.303–0.978, *p* = 0.042), and maximum spontaneous ventilation (MVV) (OR = 0.980, 95% CI: 0.964–0.995, *p* = 0.011) were significantly negatively correlated with postoperative VTE in patients with stage IA NSCLC. There was no significant difference in other variables.

**TABLE 2:** Logistic regression analysis of the risk factors for VTE in the training set

		Univariate analysis			Multivariate analysis		
Factors		OR	95%CI	*P*	OR	95%CI	*P*
Age		1.120	1.060–1.190	**<0.001**	1.095	1.006–1.193	**0.037**
Sex							
Male		Ref					
Female		1.199	0.513–2.803	0.676			
Hospital length of stay(d)		1.021	0.946–1.103	0.590			
BMI		1.058	0.937–1.195	0.360			
Surgical approach							
VATS		Ref			Ref		
Open		12.652	1.703–93.995	**0.013**	1.332	0.024–72.577	0.888
Extent of resection							
Wedge		Ref					
Segment		0.819	0.132–5.105	0.831			
Lobe		2.115	0.607–7.369	0.240			
Operation time		1.010	1.003–1.017	**0.003**	1.006	0.997–1.016	0.210
Bleeding		1.002	1.000–1.003	**0.023**	1.000	0.997–1.002	0.769
Pre‐PLT		1.004	0.997–1.011	0.239			
Pre‐APTT		0.970	0.846–1.112	0.662			
Pre‐PT		1.249	0.670–2.330	0.484			
Pre‐D‐Dimer		2.676	1.456–4.919	**0.002**	2.346	1.120–4.916	**0.024**
Post‐PLT		0.999	0.991–1.007	0.799			
Post‐APTT		1.129	0.996–1.28	0.058			
Post‐PT		1.235	0.697–2.187	0.469			
Post‐D‐Dimer		1.154	1.022–1.304	**0.021**	1.088	0.915–1.294	0.340
FEV1		0.478	0.239–0.957	**0.037**	2.996	0.307–29.218	0.345
FVC		0.554	0.303–0.978	**0.042**	0.331	0.060–1.839	0.206
MVV		0.980	0.964–0.995	**0.011**	0.990	0.957–1.024	0.565
IVD		7.466	3.169–17.592	**<0.001**	5.380	1.729–16.740	**0.004**
Module morphology							
GGO		Ref					
Subsolidity		2.265	0.819–6.263	0.115			
Solidity		1.560	0.506–4.815	0.439			
Location							
LU		Ref					
LL		0.235	0.028–1.942	0.179			
RU		1.038	0.398–2.704	0.939			
RM		0.401	0.048–3.371	0.400			
RL		0.786	0.225–2.749	0.706			
Pathological diameter		1.565	0.813–3.012	0.180			
T stage							
T1a		Ref			Ref		
T1b		1.857	0.718–4.801	0.201	1.301	0.395–4.281	0.665
T1c		3.519	1.039–11.917	**0.043**	1.018	0.171–6.068	0.984
Pathological type							
AD		Ref					
SCC		1.690	0.200–14.315	0.630			
Others		/	/	/			
LNR		1.061	1.013–1.112	**0.012**	1.063	0.994–1.137	0.073

Abbreviations: AD, adenocarcinoma; APTT, activated partial thromboplastin time; BMI, body mass index; FEV1, forced expiratory volume in one second; FVC, forced vital capacity; GGO, ground glass opacity; LNR, lymph node removal; LU, left upper lobe; LL, left lower lobe; MVV, maximal voluntary ventilation; PLT, platelet; PT, Prothrombin time; RM, right middle lobe; RL, right lower lobe; RU, right upper lobe; SCC, squamous cell carcinoma; VATS, video‐assisted thoracoscopic surgery; VTE, venous thromboembolism.

Bold values were all *P* < 0.05, indicating that the factor was statistically significant, and then these factors were included in the multivariate analysis.

All of the abovementioned significant factors were included in the multivariate logistic regression analysis. The results are shown in Table 2; age (OR = 1.095, 95% CI: 1.006–1.193, *p* = 0.037), preoperative D‐dimer (OR = 2.346, 95% CI: 1.120–4.916, *p* = 0.024) and intermuscular venous dilatation (OR = 5.380, 95% CI: 1.729–16.740, *p* = 0.004) were independent risk factors for postoperative VTE in patients with stage IA NSCLC.

### 3.3 Nomogram construction

**FIGURE 2:**
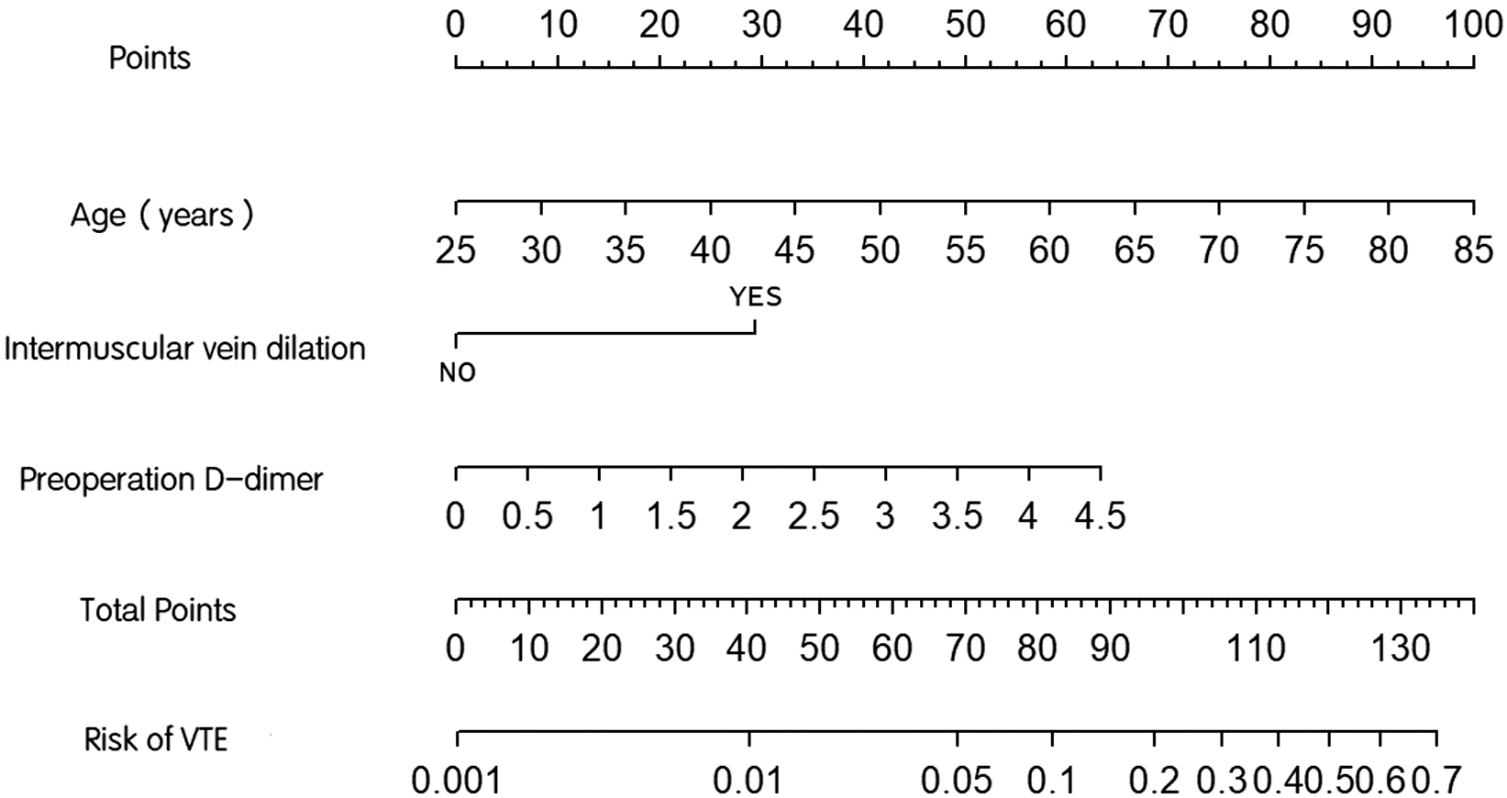
Nomogram for predicting postoperative VTE probability in patients with stage IA NSCLC. Three factors were calculated into the VTE prediction nomogram. VTE, Venous thromboembolism; NSCLC, non‐small cell lung cancer.

A nomogram was constructed from the results of multivariate logistic regression analysis of the training set (Figure 2). The nomogram showed that age had the greatest influence on postoperative VTE, followed by preoperative D‐dimer and intermuscular vein dilatation. Each parameter of each factor in these variables is assigned a score on the model, and a cumulative total risk score is finally calculated. Then, drawing a straight line down can intuitively estimate the probability of postoperative VTE for each patient.

### 3.4 Calibration and validation of the nomogram

**FIGURE 3:**
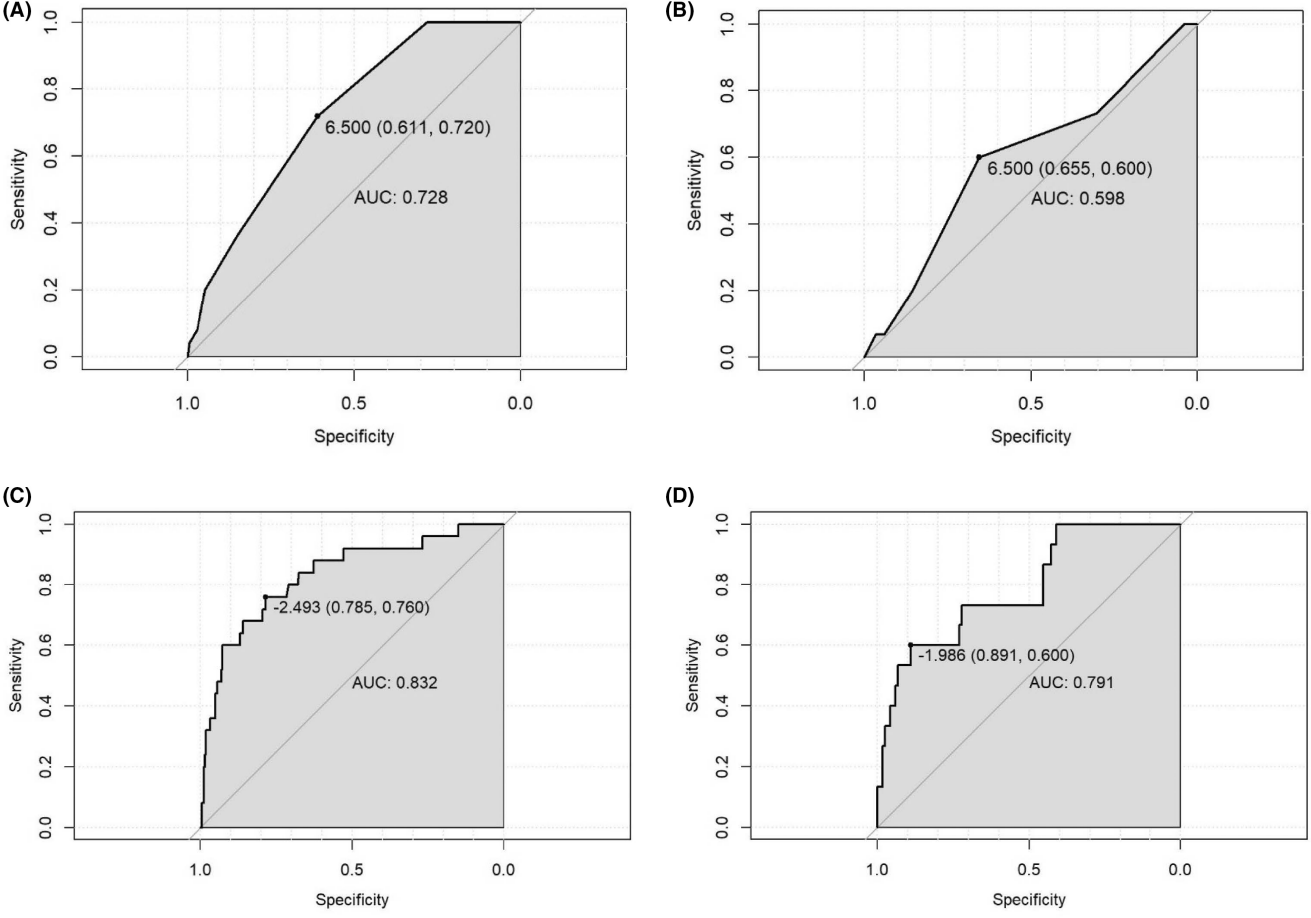
Receiver operating characteristic (ROC) curves used for differentiation in training and validation sets. (A, B)The areas under the curve (AUC) of modified caprini RAM were 0.728 (95%CI:0.639–0.816) and 0.598 (95%CI:0.440–0.755), respectively. (C, D)The areas under the curve (AUC) of the nomogram were 0.832 (95%CI, 0.732–0.924) and 0.791 (95%CI, 0.668–0.930), respectively, indicating that the model showed a better discriminative power.

**FIGURE 4:**
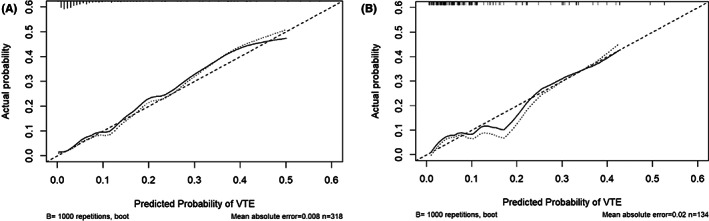
Calibration curves for training and validation sets (A, B). The x‐axis shows the model's predicted probability, and the y‐axis shows the actual probability.

Receiver operating characteristic (ROC) curves were plotted in the training and validation sets. The AUC values of the modified Caprini RAM were 0.728 (95% CI: 0.639–0.816) and 0.598 (95% CI: 0.440–0.755). The results are shown in Figure 3A and B. The AUC values of the nomogram in the training set and validation set were 0.832 (95% CI: 0.732–0.924) and 0.791 (95% CI: 0.668–0.930), respectively, which were higher than those of the modified Caprini RAM. It is suggested that the nomogram prediction model has better discriminative power, and the results are shown in Figure 3C and D. In addition, the calibration curve was applied to evaluate the agreement between the predicted probability and the true probability of the nomogram prediction model. The results are shown in Figure 4A and B. The predicted curves of the nomogram in the training set and the validation set have good fitting consistency with the real curve. The C‐index values are 0.832 and 0.790, respectively, indicating that the model has significant discrimination.

### 3.5 Clinical use

**FIGURE 5:**
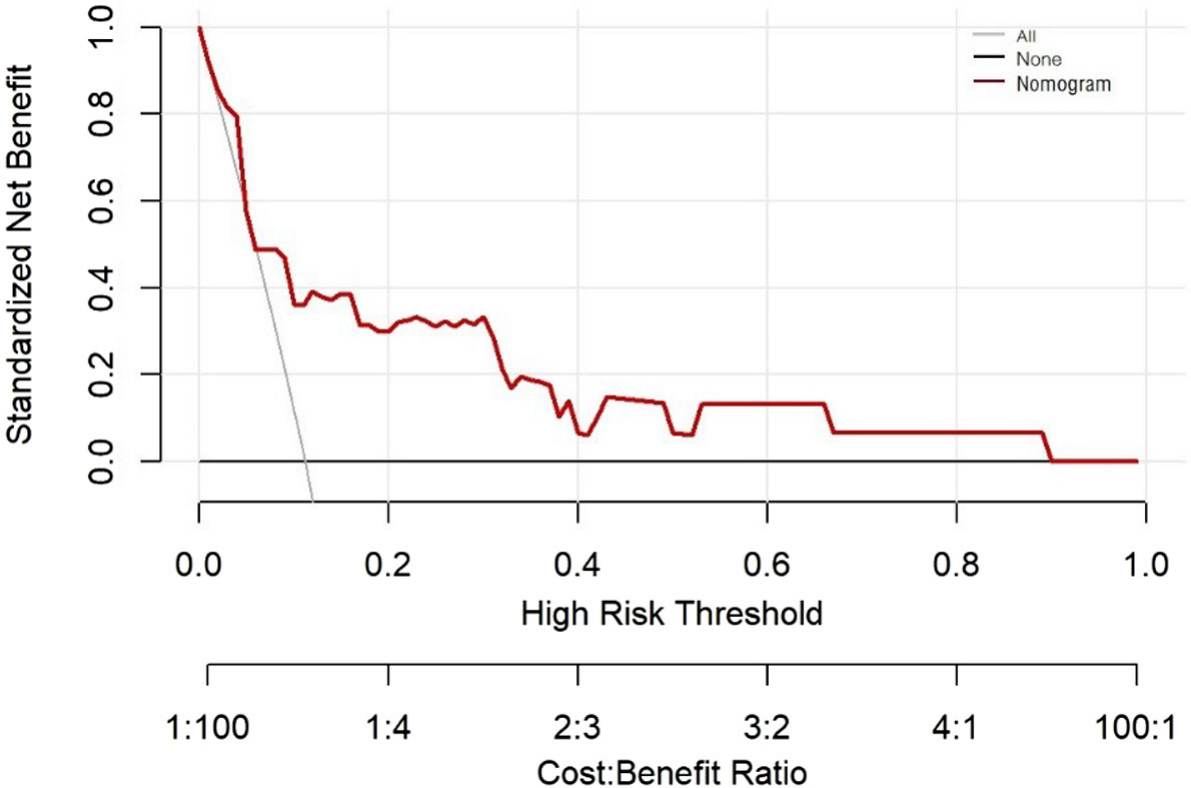
Decision curve analysis of Nomogram for predicting postoperative VTE risk in patients with stage IA NSCLC. VTE, venous thromboembolism; NSCLC, non‐small cell lung cancer.

The DCA of the nomogram in the validation set is shown in Figure 5. In the decision curve, the nomogram for predicting VTE showed more benefit than all or none if the patient's threshold probability was between 5% and 90%.

## 4 DISCUSSION

Surgery remains the mainstay of treatment for NSCLC. VTE is a common complication after surgery in patients with NSCLC, and its occurrence significantly increases patient mortality.^3^, ^4^, ^5^, ^6^ The number of patients with stage IA NSCLC undergoing thoracic surgery has increased rapidly in recent years.^26^, ^27^ However, there are few studies on the incidence and risk factors for postoperative VTE in these patients. It is necessary to better understand the risk factors for postoperative VTE in such patients. In addition, establishing a good VTE prediction model can help clinicians accurately identify high‐risk patients with VTE promptly. This study attempted to establish a reliable nomogram of 452 patients to predict postoperative VTE in stage IA NSCLC to predict the risk probability of thrombosis in these patients accurately.

The nomogram prediction model established in this study included age, preoperative D‐dimer, and intermuscular vein dilation. It has accurate prediction power, with a C‐index of 0.832, and has been internally verified. The results also confirmed that the model has a good performance of distinction. A nomogram is a method to accurately predict the probability of occurrence of each individual outcome event by integrating multiple predictors. Previous studies have confirmed that nomograms can accurately predict the risk of VTE in patients.^17^, ^21^, ^22^ Wang et al.^17^ used age, D‐dimer, body mass index (BMI), and a surgical approach to establish a nomogram to accurately predict the probability of postoperative VTE in patients with gynecological malignancies, and the C‐index was 0.721. Similarly, Zhang et al.^21^and Li et al.^22^ used a nomogram to establish a predictive model for postoperative VTE of spinal metastatic tumors and postoperative VTE of breast cancer, respectively, which can achieve individualized and accurate prediction.

It is well known that age is an important influencing factor for VTE, and the results of our study show that age is still an independent risk factor for stage IA NSCLC. The risk of postoperative VTE increased by 1.095 times for each additional 10 years of age, consistent with previous lung cancer‐related studies.^28^, ^29^, ^30^ Among them, Wang et al.^30^ showed that the incidence of postoperative VTE in patients older than 60 years was significantly higher than that in patients younger than 60 years (17.1% vs. 6.3%). In addition, in patients undergoing surgery for gynecological tumors or breast cancer, advanced age is also a high‐risk factor for postoperative VTE.^17^, ^21^, ^22^

D‐dimer is a degradation product of fibrin and is a sensitive indicator for judging fibrinolytic status and coagulation function. Several studies^28^, ^30^, ^31^, ^32^ have confirmed that D‐dimer is an independent risk predictor of VTE. The results of this study are highly consistent with those of previous studies,^9^, ^22^, ^31^, ^32^ all of which suggested that pretreatment D‐dimer elevation significantly increased the risk of VTE in patients. Tian et al. showed that the AUC value of preoperative D‐dimer in VTE after lung resection was 0.70 and suggested that patients with elevated preoperative D‐dimer should undergo VTE prophylaxis.^9^ In addition, Li et al.'s study^22^ confirmed that with an increase in the preoperative D‐dimer value to varying degrees, the risk of VTE in breast cancer patients after surgery increased by 0.831–4.036 times. A previous prospective, the single‐center study also confirmed that increased D‐dimer in patients before chemotherapy was significantly associated with a higher risk of VTE after chemotherapy.^32^ The results of our study suggest that preoperative D‐dimer elevation is an independent risk factor for stage IA NSCLC. Therefore, paying attention to the level of D‐dimer before treatment can help clinicians identify some patients with a prothrombotic state in time.

Calf intermuscular vein dilatation is also an independent factor for postoperative VTE in patients with stage IA NSCLC. Most of the patients with VTE events in this study had calf intermuscular vein thrombosis, a specific type of DVT.^33^Therefore, calf intermuscular vein dilatation was considered a risk predictor for research analysis. The calf intermuscular venous plexus has many branches, a small diameter, a thin wall, few venous valves, slow blood flow, and the absence of deep fascia and other hard tissues around it; thus, it is also prone to thrombosis. Recent studies related to lung cancer have shown that the incidence of VTE in patients with varicose veins of the lower extremities is approximately five times that of patients without varicose veins.^34^ In addition, a previous study on risk factor analysis of postoperative VTE in patients with lung cancer showed that calf intermuscular vein dilatation was an independent risk factor for postoperative VTE after lung cancer, which was consistent with the results of our study.^35^

This study shows that the modified Caprini RAM has certain limitations when evaluating postoperative VTE in patients with stage IA NSCLC, and the nomogram has excellent discriminative ability. When a nomogram predicts the probability of postoperative VTE in patients with stage IA NSCLC, it is not only convenient to apply but also has accurate and personalized results. In both datasets of this study, the AUC values of the nomogram were higher than those of the modified Caprini RAM, which had better predictive power. At present, the modified CapriniRAM is the most widely used in the field of thoracic surgery. It evaluates VTE risk in patients in the form of scores and has good risk stratification. A previous prospective study^7^ confirmed that when lung resection patients were assessed by Caprini RAM, the incidence of VTE in the low‐risk group, intermediate‐risk group, and high‐risk group was 0%, 12.3%, and 40.0%, respectively, with a good stratification effect and was validated in a recent multicenter study.^36^ However, none of the above studies analyzed patients with stage IA NSCLC, and this study only included patients with stage IA NSCLC for analysis and model establishment, which confirmed that the modified CapriniRAM has certain limitations, possibly because the model did not include some critical risk factors, such as D‐dimers and calf intermuscular vein dilation. A nomogram allows for the addition of potential biomarkers to achieve precise individualized predictions.

Our study has the following limitations. First, single‐center, retrospective studies reduce the generalizability of the model. Second, although the validation set in the study was randomly assigned, a large amount of multicenter data is still needed for external validation. Finally, patients after discharge were not considered in this study, which may underestimate the incidence of postoperative VTE in patients with stage IA NSCLC.

## 5 CONCLUSION

In summary, our study retrospectively analyzed 452 patients with stage IA NSCLC, established a nomogram based on three independent risk factors to predict the risk probability of postoperative VTE in patients with stage IA NSCLC and validated the model, confirming its good prediction performance. We believe that the application of this model will allow clinicians to accurately assess the risk of postoperative VTE in patients with stage IA NSCLC, thereby providing patients with appropriate individualized prevention and treatment strategies.

## AUTHOR CONTRIBUTIONS

Yongsheng Cai and Honghong Dong contributed to manuscript writing, acquisition of data and analysis, and interpretation of data; Jinbai Miao and Qirui Chen contributed to conception and design, revising it critically for important intellectual content. Xinyang Li and Yi Liu have been involved in figure preparation and acquisition of data. Bin Hu and Hui Li revises it critically for the manuscript; All authors gave final approval of the version to be published.

## CONFLICT OF INTEREST

The authors have no relevant financial or non‐financial interests to disclose.

## ETHICAL APPROVAL AND CONSENT TO PARTICIPATE

This study was performed in line with the principles of the Declaration of Helsinki. The studies involving human participants were reviewed and approved by the ethics committee of Beijing ChaoyangHospital Affiliated with Capital Medical University. The requirement for informed consent was waived owing to the retrospective nature of the study.

## Data Availability

The datasets generated during and/or analysed during the current study are available from the corresponding author on reasonable request.
